# Pigmentary mosaicism as a recurrent clinical manifestation in three new patients with mosaic trisomy 12 diagnosed postnatally: cases report and literature review

**DOI:** 10.1186/s12920-022-01382-x

**Published:** 2022-10-31

**Authors:** A. Martínez-Hernández, D. Martínez-Anaya, C. Durán-McKinster, V. Del Castillo-Ruiz, P. Navarrete-Meneses, E. J. Córdova, B. E. Villegas-Torres, A. Ruiz-Herrera, R. Juárez-Velázquez, E. Yokoyama-Rebollar, D. Cervantes-Barragán, A. Pedraza-Meléndez, L. Orozco, P. Pérez-Vera, C. Salas-Labadía

**Affiliations:** 1 Laboratorio de Inmunogenómica y Enfermedades Metabólicas, Instituto Nacional de Medicina Genómica SS, Ciudad de Mexico, México; 2 Laboratorio de Genética y Cáncer, Departamento de Genética Humana, Instituto Nacional de Pediatría, Ciudad de Mexico, 04530 México; 3 Departamento de Dermatología, Instituto Nacional de Pediatría, Ciudad de Mexico, México; 4 Departamento de Genética Humana, Instituto Nacional de Pediatría, Ciudad de Mexico, México; 5 Consorcio de Oncogenómica, Instituto Nacional de Medicina Genómica SS, Ciudad de Mexico, México; 6 Instituto Nacional de Medicina Genómica SS, Ciudad de Mexico, México; 7 Hospital de Especialidades Pediátrico de León, León, Guanajuato, México; 8 Hospital Central Sur de Alta Especialidad, PEMEX, Ciudad de Mexico, México; 9 Posgrado en Ciencias Biológicas, Universidad Nacional Autónoma de México, Ciudad de Mexico, México

**Keywords:** Case report, Mosaic trisomy 12, Pigmentary mosaicism, Blaschko lines, Low-level mosaicism, Uniparental disomy 12, SNP array

## Abstract

**Background:**

To date, only twenty-one cases diagnosed postnatally with mosaic trisomy 12 have been reported. The most frequent phenotypic manifestations are developmental delay, dysmorphic facial features, congenital heart defects, digital alterations, and pigmentary disorders. In the present report, detailed clinical and genetic profiles of three unrelated new patients with mosaic trisomy 12 are described and compared with previously reported cases.

**Case presentation:**

In the present report, we include the clinical, cytogenetic, and molecular description of three Mexican patients diagnosed postnatally with mosaic trisomy 12. At phenotypic level, the three patients present with developmental delay, dysmorphic facial features, congenital heart defects and skin pigmentary anomalies. Particularly, patient 1 showed unique eye alterations as bilateral distichiasis, triple rows of upper lashes, and digital abnormalities. In patient 2 redundant skin, severe hearing loss, and hypotonia were observed, and patient 3 presented with hypertelorism and telecanthus. Hyperpigmentation with disseminated pigmentary anomalies is a common trait in all of them. The cytogenetic study was carried out under the strict criteria of analysis, screening 50–100 metaphases from three different tissues, showing trisomy 12 mosaicism in at least one of the three different tissues analyzed. With SNParray, the presence of low-level mosaic copy number variants not previously detected by cytogenetics, and uniparental disomy of chromosome 12, was excluded. STR markers allowed to confirm the absence of uniparental disomy as well as to know the parental origin of supernumerary chromosome 12.

**Conclusions:**

The detailed clinical, cytogenetic, and molecular description of these three new patients, contributes with relevant information to delineate more accurately a group of patients that show a heterogeneous phenotype, although sharing the same chromosomal alteration. The possibility of detecting mosaic trisomy 12 is directly associated with the sensitivity of the methodology applied to reveal the low-level chromosomal mosaicism, as well as with the possibility to perform the analysis in a suitable tissue.

## Background

The incidence of chromosome aneuploidy in newborns is approximately 0.3%, being trisomy 13, 18, and 21 the most common abnormalities [1, 2]. Euploid/aneuploid mosaic often involves chromosomes 1, 9, 14, 16, and 21 [2]. Postnatally mosaic trisomy for chromosome 12 is considered a rare finding [2] and to our knowledge, there are only twenty-one reported cases diagnosed with mosaic trisomy 12 after birth [3–18]. At phenotypic level, mosaic trisomy 12 includes patients with an apparently normal phenotype to patients with short stature, hypotonia, microcephaly, developmental delay, dysmorphic facial features such as epicanthal folds, broad nasal bridge, and low-set rotated ears, congenital heart defects, digital alterations and pigmentary mosaicism [3, 8, 17–19].

It is known that the clinical manifestations associated with chromosomal mosaic alterations depend on the timing of the mosaicism-inducing event, the specific type of affected cells, the level of mosaicism, the chromosome involved, the distribution of abnormal cells in different tissues and the presence of UPD [5, 15, 18, 20, 21]. The probable origin of mosaic trisomy 12 can be explained by a non-disjunctional meiotic event generating a trisomic zygote, followed by mitotic trisomy rescue (mitotic correction), or by mitotic non-disjunction [18].

In the present report, we include the clinical, cytogenetic, and molecular description of three unrelated Mexican patients diagnosed postnatally with mosaic trisomy 12, with a brief description of the phenotype, and discussion of the common clinical features previously reported for this group of patients.

## Case presentation

Patients were diagnosed by the Genetics and Dermatology Departments of three different hospitals. This study was approved by the Research Ethics committee with National Commission of Bioethics registration number “CONBIOETICA-09-CEI-025-20,161,215”. Signed informed consent was obtained according to the recommendations of the Helsinki Declaration.

### Patient 1

**Fig. 1:**
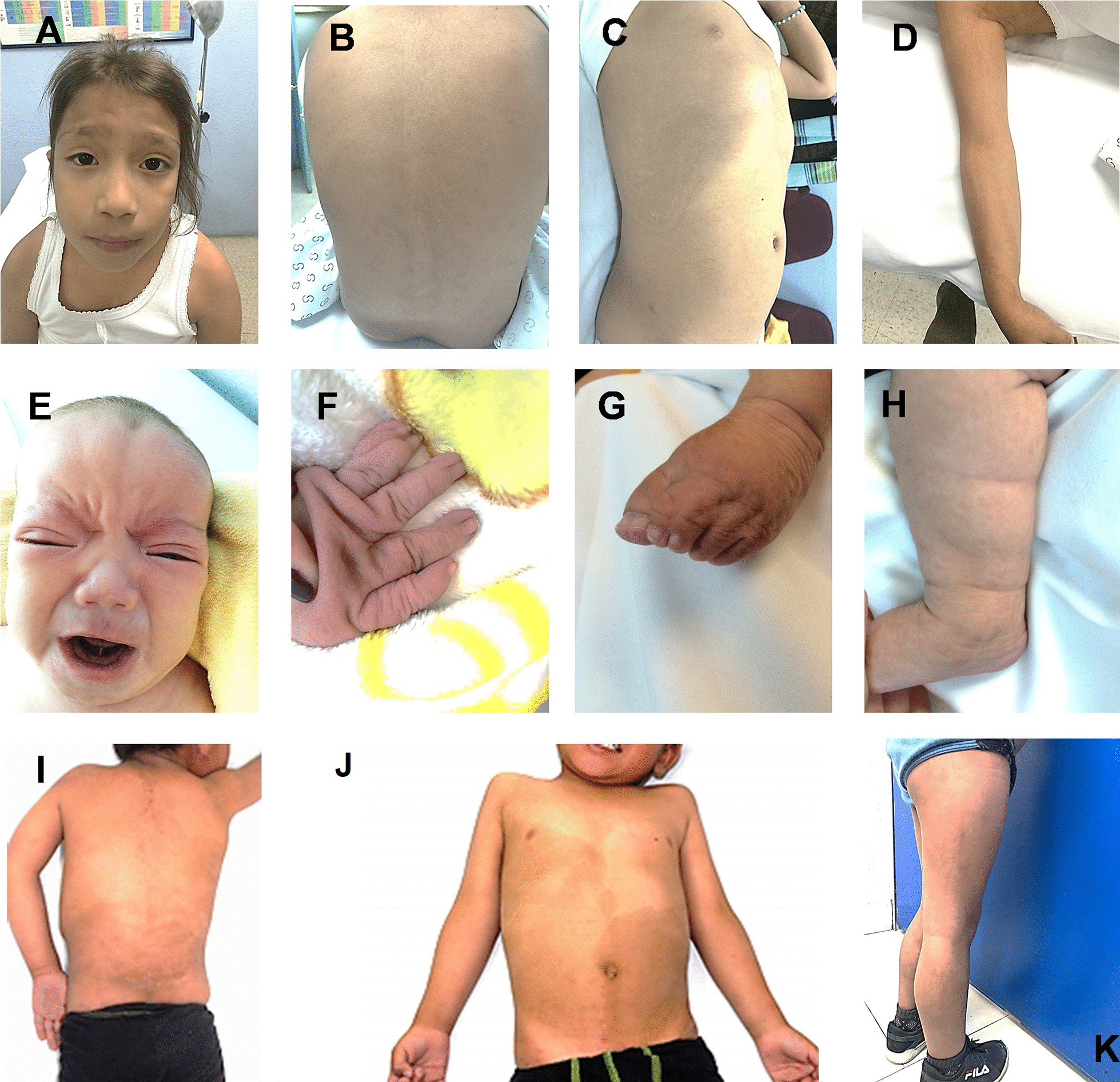
Clinical features. Patient 1 showing: (**A**) Depressed nasal bridge with midface hypoplasia; (**B**–**D**) Fine Blaschko lines with disseminated hypo and hyperpigmentation in trunk and superior limbs. Patient 2 at 15 months: (**E**) Note wide and very depressed nasal bridge, midface hypoplasia and long philtrum; (**F**) Camptodactyly of the left 2nd finger; (**G**) Complete syndactyly of the 2nd, 3rd and 4th toes and (**H**) Inferior limbs with hyperpigmented fine Blaschko lines. Patient 3 at 4 years 11 months showing: (**I**–**J**) Broad hyperpigmented BL in trunk and (**K**) Fine hyperpigmented Blaschko lines in lower limbs

An 8-year-old female was referred for dysmorphic facial features, developmental delay, and pigmentary mosaicism. She was the third child of a non-consanguineous and healthy couple with maternal and paternal age of 35 and 34 years-old, respectively. She was delivered by C-section at 39 weeks because of polyhydramnios without complications. Birth weight was 3650 g (z 0.62 SD) and length 50 cm (z −0.10 SD) (SD of three patients were obtained based Fenton growth charts according to gestational age), OFC was not available. She presented with congenital heart defects reverted by surgery at 2 years old. Physical examination showed sparse eyebrows, bilateral distichiasis and triple rows of upper lashes, eyelid ptosis and narrow eyelid fissure. Depressed and broad nasal bridge, midface hypoplasia, high-arched palate, and posteriorly rotated ears were also observed (Fig. 1A). She presented pectus excavatum and 0.5 cm umbilical hernia. Hands with bilateral brachydactyly of the 5^th^ finger and hallux valgus in both feet were also observed. In addition, pigmentary mosaicism with disseminated hypo and hyperpigmentation following fine Blaschko lines was observed (Fig. 1B–1D).

### Patient 2

A fifteen-month-old male was referred for dysmorphic facial features, severe developmental delay, and pigmentary mosaicism. The proband was the first child of a non-consanguineous healthy and young couple. He was delivered by C-section at 37 weeks of gestation because of polyhydramnios. Birth weight was 3610 g (z 1.45 SD), length 51 cm (z 1.14 SD), OFC was not available. Apgar score was 5/8. Physical examination revealed mild dolichocephaly with a prominent forehead, anterior hairline slightly resembles widow’s peak, with frontal upsweep. He showed sparse eyebrows, straight palpebral fissures, epicanthus, nystagmus, and other abnormal ocular movements. Depressed and broad nasal bridge, anteverted nares, midface hypoplasia and long philtrum were detected (Fig. 1E). He showed thin lips, downturned corners of the mouth, high palate, short uvula, and discretely cleft lip. Posteriorly rotated ears, with a skin crease in anterior earlobes, prominent antitragus, redundant skin in the back of his neck, hypoplastic nipples, diastasis recti and 1.5 cm umbilical hernia were also found, together with shawl scrotum, bilateral cryptorchidism, phimosis, sacral dimple, and a small skin tag. Hands with interphalangeal hypermobility, aberrant palmar creases, postaxial polydactyly in both hands, camptodactyly of the left 2nd finger, deep nails and complete syndactyly of the 2nd, 3rd, and 4th toes, were observed (Fig. 1F–G). He presented redundant skin and pigmentation anomalies with disseminated hyperpigmentation following fine Blaschko lines (Fig. 1H). He is now 4 years old with developmental delay, bilateral severe hearing loss, atrial septal defect, patent ductus arteriosus, hypotonia with only partial head control, erratic eye movements and pigmentary mosaicism.

### Patient 3

A 4-year-old male was referred for developmental delay, and pigmentary mosaicism. The male proband was the third child of healthy and non-consanguineous parents with maternal and paternal age of 28 and 33 years-old, respectively. Delivery occurred at 39 weeks of gestation with neonatal hypoxia and hip dislocation. Birth weight was 3100 g (z −0.58 SD), length was 51 cm (z 0.33 SD) and OFC was 36 cm (z 1.03 SD). Apgar score was 6/8. Physical examination showed arched eyebrows, hypertelorism, telecanthus, depressed nasal bridge and anteverted nares. He presented with cardiac alterations reverted by surgery at 4 months. Pigmentary mosaicism with disseminated hyperpigmentation, following broad and fine Blaschko lines in the trunk and limbs respectively, was observed (Fig. 1I–K). He has now 9 years 5 months with developmental delay and pigmentary mosaicism.

**Fig. 2:**
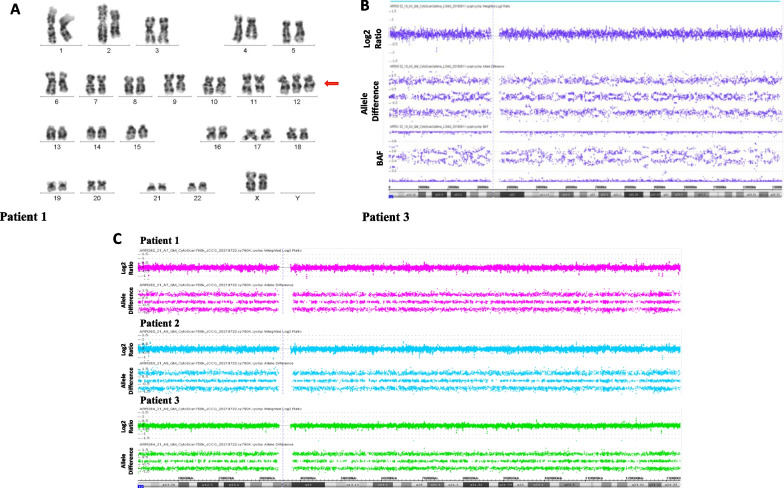
Cytogenomic analysis. (**A**) karyotype in DS of patient 1 showing only trisomy 12 cell line; (**B**) Patient 3 aCGH 400 K analysis showing chromosome 12 mosaic duplication (30%) in DS: arr[GRCh37] 12p13.33q24.33(64620_133201316) × 2 ~ 3 and (**C**) SNParray 750 K discarding copy number variants and UPD12 in PB of three patients

Cytogenetic analysis was performed in peripheral blood (PB) lymphocytes following conventional techniques and interpreted according to the International System for Human Cytogenetic Nomenclature 2020 [22]. Fresh biopsies were obtained from hypopigmented/Light skin (LS) and hyperpigmented/Dark skin (DS) areas. Fibroblasts were cultured with complete-Amniomax medium (Gibco, USA) for 10–15 days. Re-seeded cells on glass coverslips were incubated with colcemid (10 mg/ml; Gibco, USA) for 20 min and harvested to obtain metaphases. G-banded metaphases were analyzed following the same criteria as for lymphocytes. The images were captured by AXIO ImagerMI (Zeiss, Germany) microscope, using IKAROS software (Meta Systems, Germany). DS cultured fibroblasts of patient 1 revealed two cell lines, one normal and the other with a trisomy 12 in 88% of the cells. LS was normal (Fig. 2A; Table 1). Fibroblasts of LS and DS in patient 2 showed trisomy 12 in 58 and 64% of the cells, respectively (Table 1). Cytogenetic analysis showed mosaic trisomy 12 in LS fibroblasts (18%) and in DS fibroblasts (34%) of patient 3 (Table 1). In patient 3 also and only for the purpose of confirming mosaic trisomy 12, complete chromosome 12 mosaic trisomy in DS was confirmed by aCGH 400 K (Agilent Technologies, Human Genome version 19/University of California, Santa Cruz (hg19/UCSC)): arr[GRCh37] 12p13.33q 24.33(64620_133201316) × 2 ~ 3 (30%) (Fig. 2B; Table 1). Cytogenetic analysis in PB lymphocytes was normal in all three patients. The parents of the 3 patients had a normal karyotype.

**Table 1:** Overview of cytogenetic and molecular findings

Patient	Cytogenetic analysis
*1	^a^PB: 46, XX ^a^LS: 46, XX DS: mos 47, XX, + 12 [44]/46, XX [6]
2	^b^PB: 46,XY LS: mos 47, XY, + 12 [29]/46, XY [21] DS: mos 47, XY, + 12 [32]/46, XY [18]
3	^b^PB: 46, XY LS: mos 47, XY, + 12 [9]/46, XY [41] DS: mos 47, XY, + 12 [17]/46, XY [33] DS aCGH analysis: arr[GRCh38]12p13.33q24.33(64620_133201316) × 2 ~ 3

*PB* Peripheral Blood; *LS* Light Skin (hypopigmented); *DS* Dark Skin (hyperpigmented); *MMC* mitomycin C; *BLE* Bleomycin; *DEB* diepoxybutane

^a^Analysis in 100 metaphases

^b^Analysis in 50 metaphases

*Spontaneous chromosomal aberrations (chromosome and chromatid breaks) were only observed in patient 1. Induced chromosomal aberrations were evaluated following previously described criteria [23]. We observed 0.04% and 0.14% of spontaneous chromosome and chromatid breaks in LS and DS respectively. Induced chromosomal aberrations with MMC, BLE and DEB in peripheral blood were negative

**Fig. 3:**
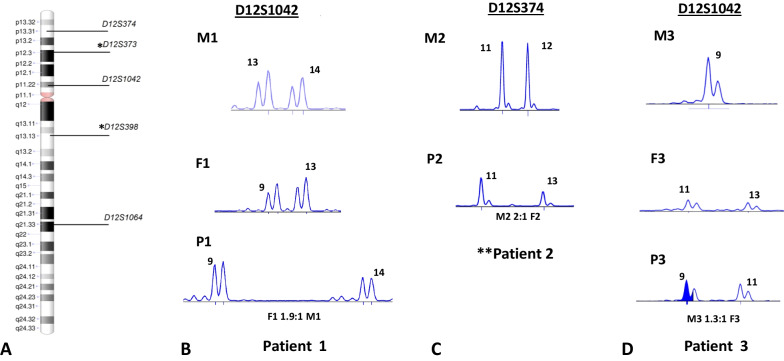
Chromosome 12 STR polymorphic markers analysis. (**A**) Ideogram showing five STR marker’s position on chromosome 12. Representative electropherograms of informative STR markers: (**B**) In patient 1, D12S1042 marker showed a dosage ratio F1 1.9:1 M1 (paternal allele F1: maternal allele M1); (**C**) In patient 2, a dosage ratio M2 2:1 F2 was observed for marker D12S374 (maternal allele M2: paternal allele F2) and (**D**) In patient 3, marker D12S1042 showed slightly different fluorescent activity with a dosage ratio M3 1.3:1 F3 (maternal allele M3: paternal allele F3). *Markers D12S398 in patients 1 and 3, as well as the marker D12S373 in patient 2, were considered non informative because of their homozygous status. **Father’s DNA sample was not suitable for molecular analysis

In order to rule out the presence of trisomy 12 as low-level mosaic in PB of the 3 patients, CytoScan™ 750 K array (ThermoFisher, USA) (NCBI GRCh37/hg19 UCSC) was performed and both, CNVs and at the same time, UPD12 were excluded in PB lymphocytes of all patients (Fig. 2C). Finally, to establish the parental origin of supernumerary chromosome 12 in trisomic cells, QF-PCR with five previously published short tandem repeats (STRs) markers was carried out on patients and parents using primers labeled with 6-FAM fluorochrome (ThermoFisher, Scientific, Foster City, CA, USA) [15]. Analysis on patient 1 showed that the extra chromosome 12 is of paternal origin (D12S1042, ratio 1.9:1) (Fig. 3A, B). We found in patient 2 that supernumerary chromosome 12 was inherited from the mother (D12S374, ratio 2:1) (Fig. 3A–C). The marker D12S1042 (ratio 1.3:1) suggested that the extra chromosome found in patient 3 was of maternal origin (Fig. 3A–D); however, for this patient, the fluorescence intensity ratio between the two alleles was below the threshold (1.8-twofold increase), making difficult to confirm trisomy. Probably, the level of mosaicism (18%) was too low to be detected by QF-PCR. It has been reported that detection of mosaicism close to 20% is possible if only biallelic ratios are observed [24]. However, heterozygosity for informative markers D12S1042 and D12S374 was retained, ruling out UPD in all patients, which was also discarded by CytoScan SNParray 750 k (Fig. 2C).

## Discussion and conclusions

Mosaic trisomy 12 remains as a rare finding in live births. Until now, only twenty-one patients have been reported [8]. Most cases have been detected prenatally (32 cases) [25–27]. Common clinical features such as developmental delay, dysmorphic facial features, musculoskeletal deformities, congenital heart defects, and pigmentary mosaicism have been described [8]. However, it is known that the clinical manifestations associated with chromosomal mosaic alterations depend on the timing of the mosaicism-inducing event, the specific type of alteration and the affected cell, the level of mosaicism and the distribution of abnormal cells in different tissues [5, 18, 21]. All these factors contribute to the clinical heterogeneity observed in these patients. In the present study, we report three Mexican patients with clinical manifestations of mosaic trisomy 12. Table 2 summarizes the clinical manifestations and genetic profile of our patients and those previously reported with mosaic trisomy 12 diagnosed postnatally.

**Table 2:** Clinical features of patients with trisomy 12 diagnosed postnatally

	Richer et al. [3]	Patil et al. [4]	Leschot et al. [11]	Von Koskull et al. [12]	English et al. [13]	*Bischoff et al. [14]			Aughton et al. [9]
						A	B	C	
*Clinical Manifestations*									
Age at diagnosis	31y	16y	9 m	Neonatal	6y	Neonatal	Neonatal	Neonatal	9 m
Developmental Delay	−	+	−	−	−	−	−	−	−
Broad forehead	-	−	−	+	−	−	−	−	−
^1^Eye alterations	-	+	−	+	−	−	−	−	−
Broad nasal bridge	−	+	−	+	−	−	−	−	−
^2^Dysmorphic ears	−	−	−	+	−	−	+	−	−
Short neck	−	−	−	+	−	−	−	−	−
^3^Other DFF	−	+	−	+	+	−	+	−	+
^4^Musculoskeletal deformities	−	+	−	+	+	−	+	−	+
^5^Congenital heart defects	−	−	−	+	+	−	−	−	−
^6^Pigmentary mosaicism	−	−	−	−	Hyper	−	−	−	−
*Genomic Analysis*									
Tissue analyzed	PB	PB Skin	AF/UCB/Placenta/Skin/ Urine sediment	AF/Placenta/ Skin	PB/Skin	CVS/ Placenta	Placenta	CVS/AF	Skin
Cytogenetic Analysis: % of trisomy 12	PB: 7	PB: 13.2% Skin: Normal	AF: 64.2 UCB: Normal Placenta: A. 31.2 B. 85 Skin: Normal Urine Sediment: A. 100 B. 77	AF: 69 Placenta: 100 Skin: 80	PB: 0.4 Skin: 11	CVS: 71 Placenta: 100	Placenta: 17	CVS: 100 AF: 100	Skin: 42
Molecular Analysis: % of trisomy 12	−	−	−	−	−	PO	PO FISH	PO	PO FISH PB: 4.2

	De Lozier- Blanchet et al. [15]	Boulard et al. [10]	Parasuraman et al. [16]	Al Hertani et al. [17]	Hong et al. [18]	Gasparini et al. [5]
*Clinical Manifestations*						
Age at diagnosis	Neonatal	Neonatal	Neonatal	Neonatal	Neonatal	2y
Developmental Delay	−	−	−	+	−	+
Broad forehead	−	−	−	+	−	+
^1^Eye alterations	+	+	−	+	+	+
Broad nasal bridge	+	−	−	−	−	−
^2^Dysmorphic ears	+	-	+	-	+	-
Short neck	-	−	−	−	+	−
^3^Other DFF	+	+	−	+	+	+
^4^Musculoskeletal deformities	+	+	−	+	−	−
^5^Congenital heart defects	+	+	+	+	−	−
^6^Pigmentary mosaicism	Hyper	−	−	Hyper	−	Hyper BL
*Genomic Analysis*						
Tissue analyzed	PB/DS	PB/Skin	AF/UCB	PB/LS/DS	PB	PB/LS/DS
Cytogenetic Analysis: % of trisomy 12	PB: Normal DS: 15 Multiple organs (40–100)	PB: Normal Skin: 80	AF: 25 UCB: 25.7	PB: Normal LS: Normal DS: 19	PB: Normal	PB: Normal LS: 28 DS: 28
Molecular Analysis: % of trisomy 12	PO FISH	−	−	−	FISH PB: 6 SNParray PB: 25	FISH

	**Hu et al. [6]				Hu et al. [7]	Hainz et al. [8]	Patient 1	Patient 2	Patient 3
	1	2	3	4					
*Clinical Manifestations*									
Age at diagnosis	Neonatal	Neonatal	Neonatal	Neonatal	Neonatal	Neonatal	8y	Neonatal	4y
Developmental Delay	+	+	+	−	−	+	+	+	+
Broad forehead	−	−	−	+	+	−	−	+	−
^1^Eye alterations	+	+	+	+	−	+	+	+	+
Broad nasal bridge	−	−	−	+	+	+	+	+	−
^2^Dysmorphic ears	+	+	+	+	+	+	+	+	−
Short neck	−	+	−	+	−	+	−	+	−
^3^Other DFF	−	+	+	+	+	+	+	+	+
^4^Musculoskeletal deformities	+	+	+	+	+	+	+	+	−
^5^Congenital heart defects	+	+	+	+	+	+	+	+	+
^6^Pigmentary mosaicism	Hyper BL	−	−	Hypo/Hyper	Hyper BL	−	Hypo/Hyper BL	Hyper BL	Hyper BL
*Genomic Analysis*									
Tissue analyzed	PB/LS/DS	PB	PB	PB	PB	PB/UUC	PB/LS/DS	PB/LS/DS	PB/LS/DS
Cytogenetic Analysis: % of trisomy 12	PB (PHA): Normal PB (PMA): 6 LS: 14 DS: Normal	PB (PHA): Normal	PB (PHA): Normal	PB (PHA): Normal	−	PB: Normal UUC: 28	PB: Normal LS: Normal DS: 88	PB: Normal LS: 58 DS: 63	PB: Normal LS: 18 DS: 34
Molecular Analysis: % of trisomy 12	FISH	SNParray PB: 0.2	CGH PB: 0.4	SNParray PB: 0.2 FISH PB_unc_: 40	FISH PB_cult_: 11.2 PB_unc_: 11.2	FISH PB: 3.5	PO SNParray	PO SNParray	PO CGH SNParray

^1^Eye alterations: Palpebral fissures: downward slant; Hypertelorism; Epicanthal folds/Epicanthus; Ptosis; Nystagmus; Telecanthus; Strabismus; Astigmatism. ^2^Dysmorphic ears: Low set/Posteriorly rotated. ^3^Other Dysmorphic Facial Features (DFF): Broad forehead; Short neck; Facial asymmetry; Depressed nasal bridge; Flat philtrum; Micrognathia; Turricephaly; Dolicocephaly; Frontal bossing; Submucous cleft palate; Macrocephaly. ^4^Musculoskeletal Deformities: Scoliosis; Hand/Feet; Atrophy muscle; Hypotonia. ^5^Congenital Heart Defects: Septal defect (VSD); Atrial septal defect (ASD); Patent ductus arteriosus (PDA). ^6^Pigmentary Mosaicism: Hypo: Hypopigmentation; Hyper: Hyperpigmentation; Hypo/Hyper: Hypopigmentation/Hyperpigmentation; BL: Pigmentation pattern following Blaschko lines. *Three different patients; **Four different patients. *y* years; *PB* Peripheral Blood; *AF* Amniotic Fluid; *UCB* Umbilical Cord Blood; *CVS* Chorionic Villus Sampling; *DS* Dark Skin; *LS* Light Skin; *PB (PHA)* Peripheral Blood culture with phytohemagglutinin*; PB (PMA)* Peripheral Blood culture with phorbol myristate acetate; *PO* Parental Origin; *CGH* Comparative Genomic Hybridization; *PB*_*cult*_ Cultured; *PB*_*unc*_ Uncultured; *UUC* Uncultured Urinary Cells

Including the patients in this study, patients with mosaic trisomy 12 have a wide spectrum of clinical manifestations. Dysmorphic facial features (20/24), cardiac alterations (15/24), developmental delay (10/24) and skin pigmentation alterations (10/24), are common features present in most previously reported cases and in the patients described herein (Table 2). All patients with trisomy 12 mosaicism, including the reported in this study, share: eye alterations (15/24), dysmorphic ears (13/24), prominent forehead (6/24), a short neck (6/24), and broad nasal bridge (8/24) (Table 2). Particularly, patient 1 showed unique eye alterations as bilateral distichiasis and triple rows of upper lashes; patient 2 showed redundant skin in the back of his neck, and patient 3 presented with hypertelorism and telecanthus. All these alterations are not common features in patients previously described in the literature (Table 2) [8]. Because a clinical hallmark of facial dysmorphism has not yet been described for patients with mosaic trisomy 12, the definition of this entity as “Mosaic Trisomy 12 Syndrome” has not been possible [6, 7, 15, 17].

Congenital heart defects, including patent ductus arteriosus, atrial septal defect, and ventricular septal defect are common features in patients with mosaic trisomy 12. These manifestations are associated mainly with genes localized on chromosome 12 short arm (p arm). These cardiac alterations are also present in patients with mosaic tetrasomy 12p or Pallister-Killian syndrome (PKS, OMIM #601803) [28, 29]; suggesting that the dosage effect of genes localized on 12p and involved in heart morphogenesis, has important implications on mosaic trisomy 12 phenotypes [6, 15, 28, 29]. Tilton et al., described some relevant genes localized on 12p associated with heart formation during embryogenesis, including *FOXM1*, *FOXJ2* and *KRAS*, and suggested that triple/quadruple doses of these genes impact cardiac development and may play a role in the presence of cardiac alterations in patients with PK-S and probably in patients with mosaic trisomy 12 [29].

At pigmentation level, all three patients reported here showed fine Blaschko lines, been hyperpigmentation with disseminated dermatosis the most frequently observed pigmentation pattern. In previous studies of patients with pigmentary mosaicism, the hypopigmentation was the most frequent type of pigmentation (ranging 50–100%) [30–34]. The herein described patients share the hyperpigmentation pattern with a cohort of cases previously described by our group, who presented this characteristic in 77% of cases [35]. In comparison, 7/21 previously reported patients with mosaic trisomy 12 showed pigmentary manifestations, described only as patchy or linear streaks [13, 15, 17]. Unfortunately, only in 3 of the total previously reported cases, a detailed description of the pigmentation pattern with disseminated dermatosis following Blaschko lines was described (Table 2) [5–7].

Cutaneous manifestations are commonly caused by somatic mosaicism, and it is known that the presence of differential skin pigmentation is related with the presence of two distinct genotypes in each type of skin [15, 35–37]. Genomic mosaicism represented by multiple non-recurring mosaic chromosomal abnormalities, and recently with mosaic single-gene variants, have been widely reported in patients with pigmentary mosaicism [30, 35, 38–42]. Chromosome 12 has at least 6 genes that are related with melanosome biogenesis: *KRT2A*, *ADAMTS20*, *WNT1*, *SILV*, *VPS33A* and *KITLG*. Copy number gains could modify the expression of any of these genes and probably generate skin pigmentary alterations [38]. It is important to highlight that the *KITLG* gene located in 12q21.32, implicated in hematopoiesis, gametogenesis, and importantly in melanogenesis, has been directly associated with pigmentary alterations. Mosaic activating *KITLG* pathogenic variants have been detected, thus extra copies of this gene produced by trisomy 12 could be associated with the pigmentary findings [38–40]. Duplication of the chromosomal region 12p12.1p11.1, which contains the *KRAS* gene, has also been associated with the presence of pigmentation abnormalities, such as cafe-au-lait spots. The increased gene dosage could deregulate the RAS/MAPK pathway, which is crucial for controlling pigmentation [41].

In our Institution, we have studied a very large and heterogeneous group of patients with pigmentary mosaicism. As part of the approach to study, in these patients with such diverse phenotypic manifestations, a very strict cytogenetic and molecular analysis is performed to find the genetic origin. As a result of this strategy of analysis, we were able to identify and diagnose the three patients reported here.

The cytogenetic study was carried out under strict criteria for screening to discard the presence of mosaicism in the three different tissues analyzed. It is important to note that the cytogenetic analysis in PB was normal in all three patients. Until now, only 4/13 patients previously reported with cytogenetic analysis in PB, showed trisomy 12 mosaicism in this tissue [3, 4, 6, 13], and in 5/5 patients in whom the trisomy 12 was analyzed by interphase FISH [6–9, 18]. Although the detection of trisomy 12 mosaicism in lymphocytes analyzing a large number of cells turns out to be efficient [43], it also has its downsides, such as: 1) The presence of mosaic restricted to specific tissues (e.g., skin); 2) PB or skin cell culturing (growth disadvantage of trisomy 12 cell lines) and 3) PB culturing with the use of phytohemagglutinin [6–8]. To avoid these problems, we emphasize the importance of looking the mosaic in other tissues such as skin, or using molecular techniques that do not require cell culturing, such as FISH and/or arrayCGH [5, 7].

Importantly, 11/24 reported patients (including patient 1 and 2, described herein) presented six or more clinical manifestations involving at least four different systems. In general, neurological, and pigmentary alterations are the ones that occurred less frequently in the above-mentioned patients; however, all three patients in the present study had neurological and pigmentary manifestations. Musculoskeletal deformities, principally hand/feet digital alterations, and cardiac alterations such as patent ductus arteriosus and atrial/ventricular septal defects are frequently observed in patients with mosaic trisomy 12, as well as in our patients (Table 2). Considering the tissues analyzed and the level of mosaicism, we observed that they are not always associated with a more severe phenotype. As shown in Table 2, three patients with all the systems affected had trisomy 12 mosaicism only in skin (DS, LS, or both; ranging 19–63%) [11], as well as patients 1 and 2 reported in this study; patients with more tissues with trisomy 12, had lesser systems altered (neither neurological nor skin) [12].

Molecular analysis with SNP array ruled out the low-level mosaic of copy number variations in PB of three patients. Same analysis detected mosaic trisomy 12 in PB of three previously reported patients [6, 18]. STR markers identified the parental origin of the extra chromosome by comparing polymorphic markers in the parents and proband [44], and with both SNP array and STR markers we also excluded UPD12 as a consequence of trisomy rescue [45].

The coexistence of disomic and trisomic cells in the same individual could be explained as follows: A non-disjunctional meiotic event generating a trisomic zygote, followed by mitotic trisomy rescue, generating an individual with a diploid biparental cell line and trisomic cell line [18]. By chance alone, two-thirds of the time a “trisomic rescue” event results in a disomic cell line with biparental inheritance, whereas one-third of the time UPD occurs [21, 46]. As known, UPD does not necessarily have a pathogenic effect, even more so when there are no imprinting regions on chromosome 12 or the presence of a pathogenic variant of a recessive disease gene, unmasked in a region of isodisomy [45]. However, UPD and its association with the presence of chromosomal aberrations such as low-level mosaic aneuploidies, can be relevant at diagnostic level [6, 21, 46–48].

In summary, trisomy 12 mosaicism is a phenotypically heterogeneous entity that occurs with very low frequency, thus, detailed clinical and cytogenomic description of these new three patients contributes relevant information to delineate more accurately a group of patients that share genetic characteristics. Our analysis strategy, looking for chromosomal abnormalities in a large number of metaphases on three different tissues, allowed us to rule out whether the alteration is confined only to a single tissue, and to accurately detect the proportion of abnormal cells. The molecular analysis discards the presence of trisomic cells in other tissues, and the presence of UPD12 originated by a non-disjunctional event. Finally, the modifications in gene expression associated with pigmentary alterations could be originated by the triple dose associated with mosaic trisomy 12. The search for biological evidence to establish these associations constitute a research challenge in patients with this entity.

## Data Availability

All the data generated during the study have been deposited in the GEO repository: The direct web link to this dataset is https://www.ncbi.nlm.nih.gov/geo/query/acc.cgi?acc=GSE212870, with accession number GSE212870.

## Abbreviations

UPD: Uniparental disomy
SD: Standard Deviation
OFC: Head circumference (occipital frontal circumference)
PB: Peripheral blood
LS: Light skin
DS: Dark skin
aCGH: Array comparative genomic hybridization
CNV: Copy number variant
QF-PCR: Quantitative fluorescent-polymerase chain reaction
STR: Short tandem repeats
6-FAM: 6-Carboxyfluorescein
SNParray: Single nucleotide polymorphism
PK-S: Pallister-Killian syndrome
FISH: Fluorescent in situ hybridization
